# Effect of prostaglandin analogues on anterior scleral thickness and corneal thickness in patients with primary open-angle glaucoma

**DOI:** 10.1038/s41598-021-90696-4

**Published:** 2021-05-27

**Authors:** Ji-Hye Park, Chungkwon Yoo, Hyun Woo Chung, Yong Yeon Kim

**Affiliations:** Department of Ophthalmology, Korea University College of Medicine, 73 Goryeodae-ro Seongbuk-gu, Seoul, 02841 South Korea

**Keywords:** Optic nerve diseases, Glaucoma

## Abstract

Prostaglandin (PG) analogues are usually prescribed as a first-line therapy in patients with glaucoma because of its once-daily dosing benefit and effective intraocular pressure (IOP) reduction. However, the mechanism of PG analogues is not completely understood. In this study, we investigated the effect of PG analogues on the anterior scleral thickness (AST) in treatment-naïve eyes with primary open-angle glaucoma using anterior segment optical coherence tomography. The AST was measured at the location of the scleral spur, 1000 μm, and 2000 μm posterior to the scleral spur and was compared before and after using the medications for 3 months and 1 year. Among 54 patients enrolled in this study, 31 patients used prostaglandin analogues and 23 patients used dorzolamide/timolol fixed combination (DTFC) drugs. There was no significant difference in untreated IOP, glaucoma severity, and baseline AST values between the two groups. While there was no significant changes in AST after using the DTFC drugs, the AST at all 3 locations showed a significant reduction in both the nasal and temporal sectors after using PG analogues for 1 year (all, *P* < 0.05). These findings suggest that the AST reduction after using PG analogues might be related with the increased uveoscleral outflow.

## Introduction

The aqueous humor leaves the eye by two main outflow pathways^1^. The conventional or trabecular outflow pathway, which accounts for approximately 70–95% of the aqueous humor outflow, consists of the trabecular meshwork, the Schlemm canal, intrascleral channels, and episcleral and conjunctival veins. In the unconventional or uveoscleral outflow, the aqueous humor exits the eye by passing through the iris root, between the ciliary muscle bundles, and then through the suprachoroidal and scleral tissues^2^. The uveoscleral outflow pathway is responsible for 5–30% of the aqueous humor outflow, with a decline in the contribution with age^3^. The cholinergic agonists are known to reduce the uveoscleral outflow, whereas the cycloplegic, adrenergic agents, and prostaglandin analogues increase this outflow^1^.

Currently, there are several different classes of glaucoma medications that lower the intraocular pressure (IOP) by enhancing the aqueous outflow and/or by reducing the aqueous production^4^; prostaglandin (PG) analogues, β-adrenergic receptor antagonists, adrenergic receptor agonists, carbonic anhydrase inhibitors, cholinergics and rho-kinase inhibitors. Among these topical glaucoma medications, prostaglandin analogues are usually prescribed as a first-line therapy in patients with glaucoma because of its once-daily dosing benefit and effective IOP reduction^5–7^. However, the mechanism of prostaglandin analogues is not completely understood and previous studies have reported inconsistent results about its mechanism of action^8^. Although several investigators^9–11^ demonstrated the improvement of the trabecular outflow facility after using prostaglandins, most animal and human studies reported that the ocular hypotensive effect of prostaglandins are not explained by reducing the aqueous production or increasing the conventional aqueous outflow, but by enhancing the uveoscleral aqueous outflow^8^. After prostaglandins bind and activate the prostaglandin receptors in the ciliary muscle, iris root, and sclera, they induce matrix metalloproteinase (MMP) activity^12,13^ and alter numerous extracellular matrix (ECM) components in these structures^14,15^, resulting in the reduction of hydraulic resistance to the aqueous movement, consequently reducing the IOP^16–18^. Therefore, the structures involved in the uveoscleral outflow may show histologic or structural changes after applying prostaglandins topically.

Recently, anterior segment imaging modalities have evolved and numerous studies have investigated the appearance of the microstructures of the aqueous outflow pathway^19–21^. In particular, several studies have scanned the corneoscleral limbus by anterior segment optical coherence tomography with enhanced depth imaging (EDI OCT) and showed the usefulness of EDI OCT for visualizing the sclera clearly and measuring the anterior scleral thickness (AST)^22–24^. Although histologic analyses have revealed the presence of transscleral fluid flux through the scleral stroma^25^ and increased permeability of the human sclera exposed to PG analogues^16,18^, the in vivo effect of PG on the human sclera is relatively unknown. Therefore, we evaluated the effect of topical prostaglandin analogues on the AST in treatment-naïve primary open-angle glaucoma (OAG) patients using EDI OCT.

## Results

A total of 65 treatment-naïve patients with OAG were consecutively enrolled in this study. Thirty-eight of them were prescribed PG analogues, and 27 were prescribed a dorzolamide/timolol fixed combination (DTFC) drug. Among the subjects, 11 subjects were excluded (7 and 4 subjects in the PG and DTFC group, respectively) because of following reasons; follow up loss (4 subjects), poor image quality (5 subjects), and medication change during follow-up (2 subjects). Finally, 54 subjects (31 and 23 subjects in the PG and DTFC group) were included for final statistical analysis (Table 1). Among the subjects in the PG group, 16, 13, and 2 subjects used latanoprost, bimatoprost, and tafluprost eyedrop, respectively. Although the patients in the PG group were younger than those in the DTFC group, there was no significant difference in the untreated IOP, axial length, visual field (VF) parameters, retinal nerve fiber layer (RNFL) thickness, and AST between the two groups. The measurements of the AST showed good intra- and inter-observer agreement (intraclass correlation coefficient, ICC, 0.956 ~ 0.992). Table 2 shows the association between the AST and other ocular factors in all subjects. Untreated IOP and central corneal thickness was positively correlated with the AST at some measurement points (*P* < 0.05).

**Table 1:** Baseline characteristics of the subjects.

	Total subjects (n = 54)	Prostaglandins (n = 31)	DTFC (n = 23)	*P* value^a^
Age (yrs)	51.35 ± 13.27	55.18 ± 13.44	45.81 ± 11.08	0.004
Untreated IOP (mmHg)	17.9 ± 3.8	17.7 ± 3.6	18.1 ± 4.0	0.661
Axial length (mm)	24.40 ± 1.43	24.26 ± 1.28	24.63 ± 1.64	0.332
Spherical equivalent (D)	− 1.69 ± 2.74	− 1.54 ± 2.49	− 1.95 ± 3.16	0.694^b^
CCT (μm)	526.05 ± 37.74	523.31 ± 37.32	530.70 ± 38.83	0.238^b^
ACD (mm)	3.39 ± 0.40	3.39 ± 0.44	3.39 ± 0.33	0.971
MD (dB)	− 5.07 ± 4.20	− 4.67 ± 3.53	− 5.74 ± 5.16	0.610^b^
PSD (dB)	5.01 ± 3.83	4.58 ± 3.19	5.74 ± 4.71	0.475^b^
VFI (%)	89.27 ± 11.51	90.54 ± 9.29	87.13 ± 14.51	0.759^b^
RNFL thickness (μm)	82.40 ± 15.43	83.79 ± 14.48	80.04 ± 17.00	0.360
**AST (μm)**				
Nasal sector				
Scleral spur (SS)	799.12 ± 65.70	801.63 ± 62.89	795.14 ± 71.28	0.720
1000 μm from SS	671.89 ± 57.49	663.54 ± 55.24	685.18 ± 59.76	0.169
2000 μm from SS	682.21 ± 50.74	672.56 ± 48.18	697.14 ± 52.06	0.076
Temporal sector				
Scleral spur (SS)	883.67 ± 63.75	877.56 ± 64.60	887.86 ± 60.87	0.694
1000 μm from SS	714.36 ± 66.10	705.12 ± 59.36	720.30 ± 71.67	0.619
2000 μm from SS	730.67 ± 71.90	724.43 ± 76.12	732.89 ± 60.94	0.882

*DTFC* dorzolamide/timolol fixed combination, *IOP* intraocular pressure, *CCT* central corneal thickness, *ACD* anterior chamber depth, *MD* mean deviation, *PSD* pattern standard deviation, *VFI* visual field index, *RNFL* retinal nerve fiber layer, *AST* anterior scleral thickness.

^a^Independent t-test, ^b^Mann-Whitney U test.

**Table 2:** Correlation between the anterior scleral thickness (AST) and other ocular factors.

	AST at Nasal sector			AST at Temporal sector		
	Scleral spur (SS)	1000 μm from SS	2000 μm from SS	Scleral spur (SS)	1000 μm from SS	2000 μm from SS
Age	− 0.156	− 0.217	− 0.161	− 0.060	− 0.229	− 0.091
Untreated IOP	0.270*	0.236	0.362**	0.165	0.266*	0.200
Axial length	− 0.151	− 0.050	− 0.099	− 0.043	− 0.079	− 0.184
CCT	0.027	0.144	0.113	0.199	0.328*	0.294*
ACD	− 0.099	− 0.062	− 0.099	− 0.114	− 0.072	− 0.166
MD	− 0.027	− 0.066	− 0.054	0.031	− 0.047	0.065
PSD	0.048	0.062	− 0.016	− 0.105	− 0.084	− 0.064
VFI	− 0.081	− 0.069	− 0.20	− 0.009	− 0.080	0.037
RNFL thickness	− 0.059	− 0.151	− 0.119	− 0.084	− 0.019	0.019

*IOP* intraocular pressure, *CCT* central corneal thickness, *ACD* anterior chamber depth, *MD* mean deviation, *PSD* pattern standard deviation, *VFI* visual field index, *RNFL* retinal nerve fiber layer.

Spearman correlation test, *P < 0.05, **P < 0.01.

**Figure 1:**
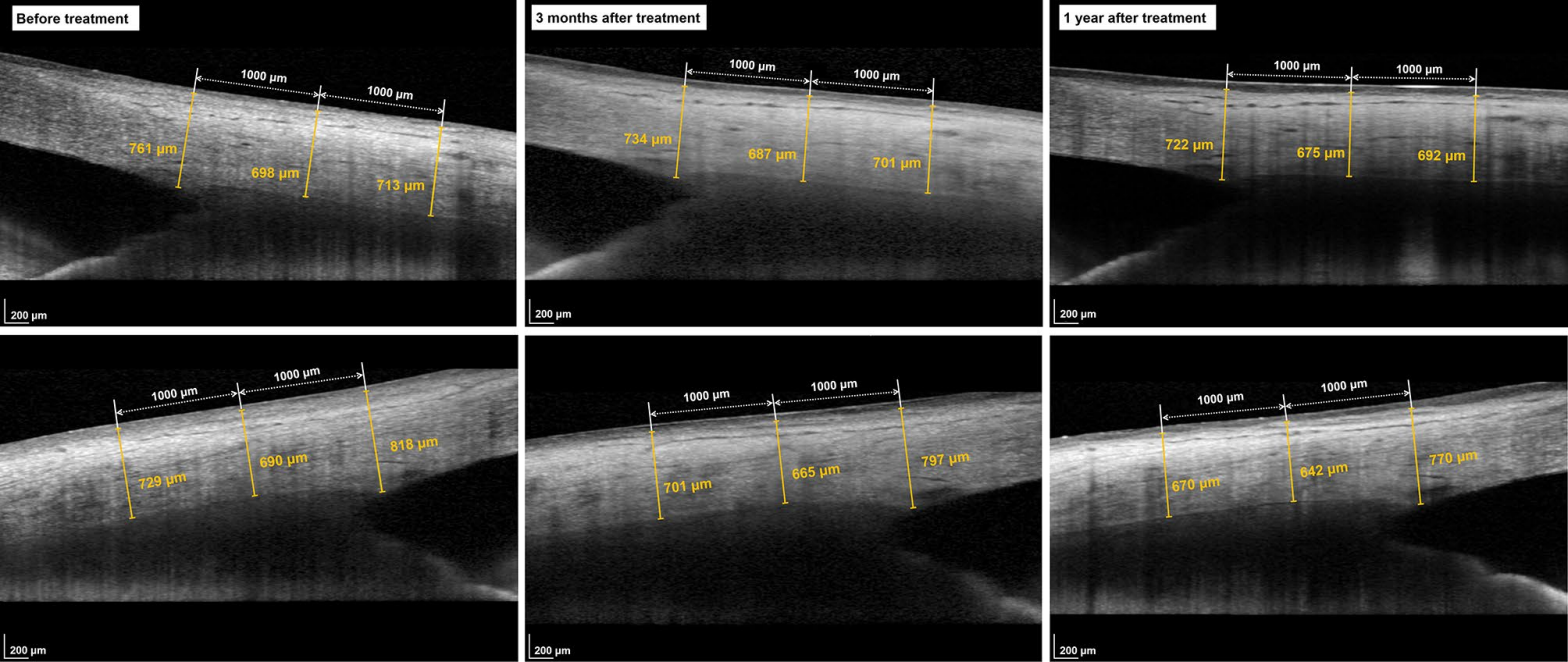
Representative case demonstrating anterior scleral thickness reduction after using prostaglandin analog for 1 year. (Top, nasal sector; Bottom, temporal sector).

The IOP, central corneal thickness (CCT), and AST measurements before and after using the IOP-lowering medications are demonstrated in Table 3. The IOP was reduced significantly after the use of the medications in both groups. In addition, the CCT showed significant thinning after using PG analogues (*P* < 0.001). However, the CCT did not change in the DTFC group. After using DTFC eye drops, the AST showed no significant changes in both the nasal and temporal meridians (*P* > 0.05). However, the AST at both meridians significantly was reduced when PG analogues were used for 1 year, except for the AST at 2000 μm posterior to the scleral spur in the nasal sector which showed a borderline significance (P = 0.044). In the subgroup analysis, subjects in the PG group were divided into two groups according to their amount of IOP reduction percentages at 1 year: the small (IOP reduction percentage ≤ 20%) and large (IOP reduction percentage > 20%) IOP reduction groups. The baseline ocular variables and AST changes were compared between the two groups (Table 4). Although only the difference in AST change at 2000 μm posterior to the scleral spur in nasal sector showed a statistical significance (*P* = 0.049), the amount of AST changes were larger in the large IOP reduction group compared to the small IOP reduction group. However, the baseline IOP, CCT, axial length, mean deviation (MD), and RNFL thickness were similar between the two groups. Figure 1 shows a representative case of AST reduction after using PG analogue for 1 year.

**Table 3:** Comparison of the anterior scleral thickness before and after using topical medications.

	Prostaglandin (n = 31)						DTFC (n = 23)					
	Before	3 month	1 year	*P* value^a^	*P* value^b^	*P* value^c^	Before	3 month	1 year	*P* value^a^	*P* value^b^	*P* value^c^
IOP (mmHg)	17.7 ± 3.6	13.8 ± 2.6	13.7 ± 2.8	< 0.001	< 0.001	< 0.001	18.1 ± 4.0	15.4 ± 3.1	15.2 ± 3.3	< 0.001	0.001	< 0.001
CCT (μm)	523.31 ± 37.32	517.2 ± 35.72	512.17 ± 35.82	< 0.001	0.020	< 0.001	530.70 ± 38.83	530.35 ± 39.05	527.00 ± 53.25	0.394	0.864	0.232
**Anterior scleral thickness (μm)**												
Nasal sector												
Scleral spur (SS)	801.63 ± 62.89	796.89 ± 61.65	779.80 ± 52.20	0.001	0.382	< 0.001	795.14 ± 71.28	805.50 ± 74.18	810.53 ± 73.88	0.134	0.078	0.530
1000 μm from SS	663.54 ± 55.24	654.66 ± 59.05	647.08 ± 50.45	0.008	0.057	0.004	685.18 ± 59.76	690.00 ± 70.84	692.87 ± 58.77	0.157	0.404	0.255
2000 μm from SS	672.56 ± 48.18	670.29 ± 51.21	656.04 ± 52.53	0.048	0.559	0.044	697.14 ± 52.06	702.09 ± 64.11	700.60 ± 55.76	0.221	0.394	0.201
Temporal sector												
Scleral spur (SS)	877.56 ± 64.60	867.59 ± 68.95	840.45 ± 60.36	0.001	0.093	0.001	887.86 ± 60.87	888.82 ± 72.16	910.44 ± 62.67	0.354	0.866	0.299
1000 μm from SS	705.12 ± 59.36	695.39 ± 59.04	671.62 ± 53.48	0.003	0.088	0.003	720.30 ± 71.67	727.35 ± 70.19	742.13 ± 69.72	0.266	0.341	0.119
2000 μm from SS	724.43 ± 76.12	710.20 ± 62.60	680.28 ± 61.85	0.013	0.026	0.013	732.89 ± 60.94	747.68 ± 66.89	744.69 ± 65.63	0.125	0.043	0.378

*DTFC* dorzolamide/timolol fixed combination, *IOP* intraocular pressure, *CCT* central corneal thickness.

^a^Repeated measures ANOVA.

^b^Paired t-test (pairwise comparison between baseline and 3 month values, *P* < 0.025 is considered statistically significant).

^c^Paired t-test (pairwise comparison between baseline and 1 year values, *P* < 0.025 is considered statistically significant).

**Table 4:** Comparison of two groups according to the IOP reduction percentage in prostaglandins medication group.

	Small IOP reduction group (n = 15)	Large IOP reduction group (n = 16)	*P* value^a^
Baseline IOP (mmHg)	17.6 ± 3.5	17.5 ± 3.5	0.938
IOP at 1 year (mmHg)	15.8 ± 2.4	13.2 ± 3.5	0.010
CCT (μm)	528.00 ± 34.27	514.82 ± 44.03	0.363^b^
AXL (mm)	24.22 ± 1.34	24.37 ± 1.33	0.317
MD (dB)	− 5.45 ± 4.27	− 4.45 ± 2.76	0.631^b^
RNFL thickness (μm)	79.38 ± 16.90	87.06 ± 11.95	0.140
IOP reduction percentage (%)	6.76 ± 11.79	29.27 ± 7.37	< 0.001
△CCT (μm)	14.56 ± 16.59	7.41 ± 11.79	0.382^b^
**△AST(μm)**			
Nasal sector			
Scleral spur (SS)	15.33 ± 34.52	31.92 ± 19.14	0.147
1000 μm from SS	7.67 ± 28.25	26.23 ± 23.65	0.087
2000 μm from SS	0.75 ± 38.38	31.00 ± 34.45	0.049
Temporal sector			
Scleral spur (SS)	25.10 ± 31.81	37.20 ± 39.94	0.463
1000 μm from SS	26.18 ± 41.75	39.20 ± 48.48	0.516
2000 μm from SS	27.20 ± 43.82	31.25 ± 47.40	0.853

*IOP* intraocular pressure, *CCT* central corneal thickness, *AXL* axial length, *MD* mean deviation, *RNFL* retinal nerve fiber layer, *AST* anterior scleral thickness.

^a^Independent t-test, ^b^Mann-Whitney U test.

## Discussion

Previous studies investigating the effect of PGs on the sclera are limited and most were conducted by in vitro experiments or by invasive methods^8,12,15–18^. To the best of our knowledge, this study is the first to report on the in vivo measurement of AST before and after using topical PG medications. The present study demonstrated that the AST decreased significantly when PG was used for 1 year in treatment-naïve patients with OAG. In addition, the CCT showed significant thinning in the PG group. However, the AST and CCT did not show significant changes when a DTFC drug was administered in eyes with OAG.

Previous studies have reported scleral changes after using PG analogues^8,12,15–18^. Gaton et al.^12^ have demonstrated that when topical PG was administered in 4 monkey eyes, the MMP immunoreactivity significantly increased in the ciliary muscle, iris root, and sclera. In addition, the reduction of collagen type I and collagen type III immunoreactivity were also noted in the ciliary muscle and the adjacent sclera following topical PG treatment^15^. Increased MMPs and reduced collagen density in the sclera may alter the scleral permeability. In fact, it has been reported that the permeability of the sclera increased when PG was administered to the human sclera in vitro^16–18^. Based on the previous in vitro studies, it can be deduced why the AST and IOP decreased after PG use. Since topical administration of PG reduced the collagen type I and collagen type III immunoreactivity, it may have induced the reduction of collagen type I density, which is the predominant type of collagen in the sclera, accounting for about one half of the total dry weight of collagen. This reduction of collagen density in the sclera may have caused a decrease in the scleral thickness. This compaction of extracellular matrix may have affected the transscleral permeability, and the enhanced transscleral permeability may have lowered the uveoscleral outflow resistance, resulting in IOP reduction.

However, the reason for the regional difference in the AST changes in the PG group, which exhibited a borderline reduction at the location of 2000 μm posterior to the scleral spur in the nasal sector, needs to be explained. First, a possible explanation is that the nasal and temporal sector may contribute differently on the uveoscleral outflow. The anterior–posterior length of the ciliary body in the adult eye ranges from 4.6 to 5.2 mm nasally to 5.6 to 6.3 mm temporally, showing a longer ciliary body in the temporal sector^1^. In addition, the baseline AST at the temporal sector was thicker compared to that at the nasal sector. Since the ciliary body and sclera are the structures involved in uveoscleral outflow, the difference in length of the ciliary body and the thickness of the anterior sclera between the nasal and temporal sectors might have influenced the scleral changes after PG medication use. Second, histologic studies have reported that the fluid flux through the sclera has two routes; fluid flux through the scleral stroma, as well as through narrow spaces around penetrating nerves and blood vessels^25^. Since the perforating blood vessel and nerves are rarest in the temporal sclera^26,27^, the transscleral fluid flux in this area may mainly rely on the pathway through the scleral stroma. Therefore, the scleral stroma, which might be correlated with the scleral thickness, in the temporal sector might have shown more dramatic changes after using topical PG. However, these aforementioned hypotheses need to be proven in further studies.

Previous studies have reported the association between the scleral thickness and other ocular factors. Woodman-Pieterse et al.^24^ have investigated the effect of accommodation on the AST and reported a reduction of the AST with accommodation, particularly in patients with myopia. Oliveira et al.^28^ used UBM to evaluate the relationship between the AST and axial length and demonstrated that the AST was thicker when the axial length was longer. In line with the previous study, the present study showed that the axial length was positively correlated with the AST at the temporal sector. However, other studies have failed to report an association between the axial length and AST^23,29,30^. Such conflicting outcomes among the studies may be attributed to the differences in the methodology or the patients’ characteristics. Also, there are studies reporting inconsistent results about the association between the CCT and AST^28,29^. Oliveira et al.^28^ reported that CCT was not related to AST in his study of 140 subjects, including 85 patients with glaucoma. In contrast, in this study of treatment-naïve patients with OAG with mean IOP 17.7 mmHg, the AST showed a positive correlation with the CCT. Further, Yoo et al.^29^ and Mohamed-Noor et al.^31^ have also found a positive correlation between the CCT and AST, but only in patients with normal-tension glaucoma (NTG). They have suggested the possibility of patients with NTG having a thinner and weaker external coat, and this weak external coat, namely the lamina cribrosa of the optic disc, may make the eye susceptible to glaucomatous damage. Further, Park et al.^32^ reported a significantly thinner subfoveal scleral thickness in NTG patients compared to high-tension POAG patients. Taken together, it can be assumed that there may be a potential relationship between the scleral thickness and the development of glaucomatous optic neuropathy.

The present study had some limitations. First, the sample size was relatively small, and an uneven number of subjects were enrolled in the two groups. This may have introduced a selection bias. Second, only the nasal and temporal sectors were evaluated. Third, only the relatively short-term effect of PG medications on AST have been evaluated. However, previous histologic studies have reported that remodeling of the extracellular matrix within the sclera and the resulting increase in the scleral permeability occur within only 3 days after drug instillation^12,15–18^. Therefore, the use of topical medication for 3 months would have been able to evaluate the effect of PGs on the anterior scleral tissue. Finally, the change in the AST may not be directly related to the increased uveoscleral outflow and IOP reduction. However, the strength of the present study is that we explored non-invasively the effect of topical PG analogues on the anterior scleral thickness measurement using EDI OCT.

In conclusion, prostaglandin analogues reduced the anterior scleral thickness in patients with open-angle glaucoma. The reduction of collagen density and the compaction of ECM may cause the reduction of the anterior scleral thickness. The reduction of anterior scleral thickness may be related with the IOP-lowering effect of prostaglandins.

## Methods

This prospective, observational study was approved by the Institutional Review Board of the Korea University Ansan Hospital. The study was conducted according to the tenets of the Declaration of Helsinki. Written informed consent was obtained from all subjects prior to study enrollment.

### Participants

Treatment-naïve patients with OAG who were first diagnosed at Korea University Ansan Hospital and had been prescribed either PG analogues or DTFC drugs were eligible for inclusion in this study. To investigate the effect of prostaglandin medication on AST changes, patients who were prescribed DTFC drugs during the same study period were selected as controls. The drug choice was based on a single physician’s judgement or on the patients’ preference. Primary open-angle glaucoma diagnosis was based on glaucomatous optic nerve head (ONH) changes, reproducible glaucomatous VF defects, and open anterior chamber angle on static gonioscopy. The following were defined as glaucomatous ONH changes: (1) focal or diffuse neuroretinal rim thinning; (2) localized notching; or (3) RNFL defects. Glaucomatous VF defects were identified when two of the following criteria were present on Swedish Interactive Threshold Algorithm (SITA) 24–2 Humphrey VF testing: (1) glaucoma hemifield test results outside normal limits; (2) a cluster of three or more non-edge, contiguous points on the pattern deviation plot, not crossing the horizontal meridian with < 5% probability of being present in age-matched healthy individuals (one of which was < 1%); and (3) pattern standard deviation < 0.05. If both eyes were eligible for inclusion in the study, we selected the eye with the lower MD value from each patient for analysis.

Patients were excluded if: (1) best-corrected visual acuity less than 20/40; (2) previous ocular surgery, except uncomplicated cataract surgery more than 1 year before recruitment; (3) presence of retinal disease; (4) refractive error exceeding spherical equivalent of 6 diopters or astigmatism of 3 diopters; or (5) invisible trabecular meshwork in any quadrant on static gonioscopy.

Each patient underwent a complete ophthalmic examinations, which included best-corrected visual acuity assessment, refractive error measurement, slit lamp biomicroscopy, Goldmann applanation tonometry, gonioscopy, CCT evaluation using a non-contact specular microscope (SP-2000p; Topcon, Tokyo, Japan), axial length measurement using an IOLMaster (Carl Zeiss Meditec, Jena, Germany), Humphrey VF testing using the SITA 24–2 test (Zeiss-Humphrey, San Leandro, California, USA), and dilated 30-degree stereoscopic photography and 50-degree red-free photography using a Zeiss FF 450 plus IR camera (Carl Zeiss Meditec Inc., Dublin, California, USA).

### Enhanced depth imaging optical coherence tomography

**Figure 2:**
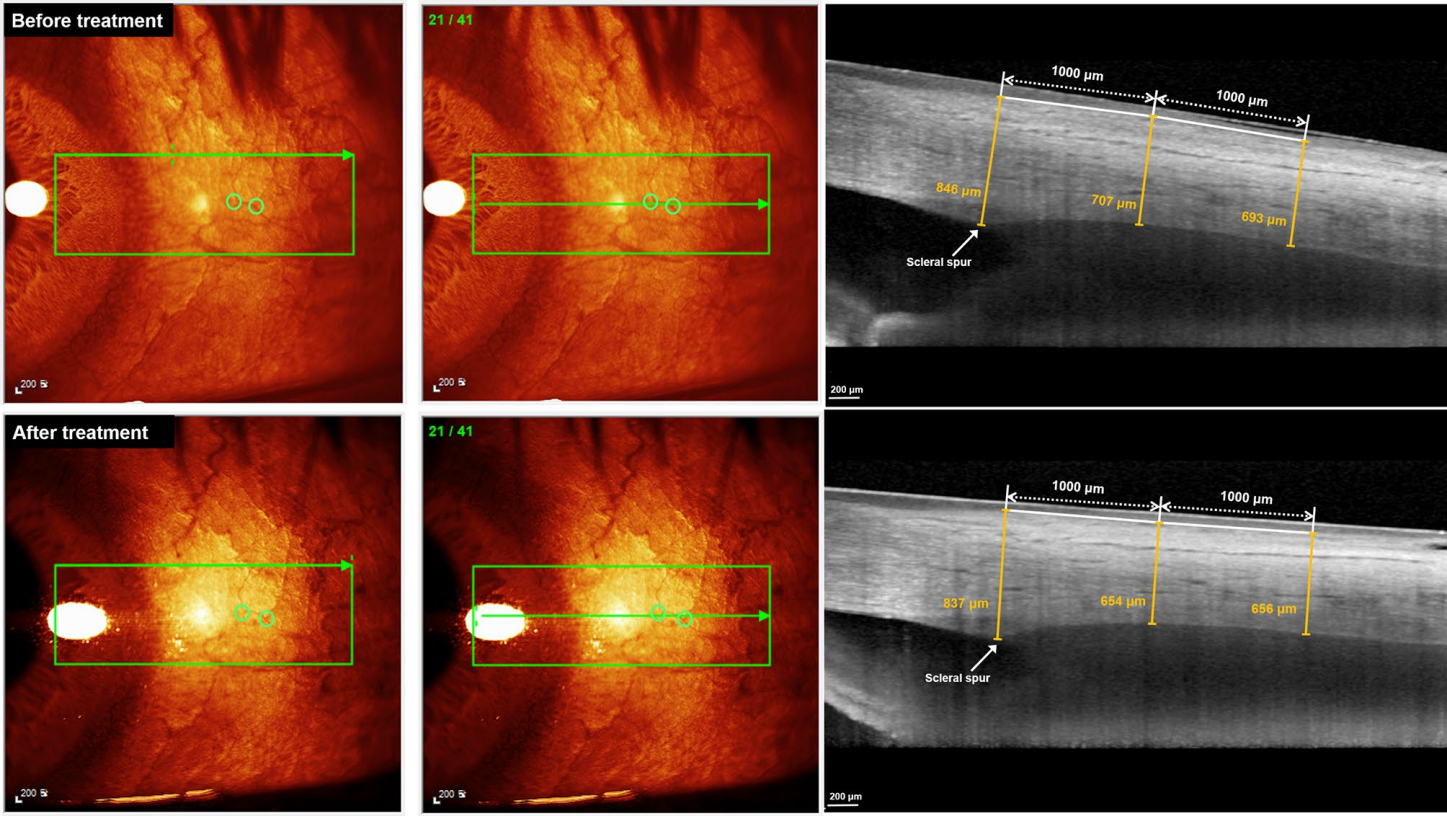
Enhanced Depth Imaging Optical Coherence Tomography B-Scans Before (Top) and After (Bottom) Administration of Topical Intraocular Pressure Lowering Agent. (Left, Middle) The conjunctival vessels were used as landmarks to compare the same B-scans before and after using the topical medications. (Right) The anterior scleral thicknesses were measured at the location of scleral spur, 1000 μm and 2000 μm posterior to the scleral spur.

A single examiner (JHP), blinded to the patients’ glaucoma status and the medication, performed the anterior segment module of spectral-domain OCT (Heidelberg Spectralis OCT, Heidelberg Engineering, Heidelberg, Germany) before and 3 months and 1 year after using the medications. Images of the nasal and temporal corneoscleral limbus were obtained with serial horizontal EDI B-scans. The instrument utilizes a super luminescent diode with central wavelength of 870 nm for OCT imaging, and measurements were set to image a 20 × 5-degree rectangle (41 EDI OCT B-scans; interval between scans, 69 μm) in the sclera mode. At each measurement session, the conjunctival vessels and iris anatomy were used as landmarks to scan the same limbal area before and after using the IOP-lowering agents. After acquisition of two volumetric scans, the conjunctival vessels and iris anatomy were carefully reviewed and used as landmarks to compare the overlapping region before and after IOP-lowering agents' usage (Fig. 2).

### Measurement of anterior scleral thickness

Patients with an incomplete set of EDI OCT B-scans or with poor quality scans in which the AST could not be reliably measured were excluded from the analysis. The AST was measured in each scan in the overlapping region between the three sets of volumetric scans (Fig. 2). The AST was measured manually using the built-in calipers of the software provided in the OCT instruments at three sites (sclera spur, 1000 μm, and 2000 μm posterior to the scleral spur) in the nasal and temporal meridians by a single blinded observer. The location of the scleral spur was selected as the point where there was a change in the curvature of the inner surface of the angle wall, often presenting as an inward protrusion of the sclera^33,34^. The first high reflective tissue signal of the episclera was considered to be the outer limit of the AST, and the interface between the sclera (highly reflective) and ciliary body (less reflective) was considered the inner limit. By measuring the AST, inter-observer variability and intra-observer reproducibility were assessed in randomly selected 20 images of 20 eyes. Intra- and inter-observer agreements in the measurements were assessed using the intraclass correlation coefficient.

### Statistics

A pilot study revealed that the standard deviation of AST difference at the temporal sector before and after using prostaglandin analogues was 54.52 μm. A sample size calculation, using G*Power software (version 3.1.9.2; Universität Kiel Dusseldorf, Germany) with α = 0.05, determined that 30 patients would be required to detect an AST difference of > 30 μm before and after using medication at a standard deviation of 54.52 μm with a power of 80%. Assuming a dropout rate of 20%, 38 patients were needed to be recruited into this study.

The SPSS software (version 21.0; SPSS, Chicago, IL, USA) was used to perform the statistical analysis. The normality of distribution was verified using the Shapiro–Wilk normality test. The patients were divided into two groups according to the medications, and only one eye per subjects were used for the analysis. The baseline characteristics, axial length, CCT, VF parameters, and IOP differences between the two groups were compared using either the independent t-test or the Mann–Whitney U test, as appropriate. The repeated measures analysis of variance (ANOVA) was used to compare AST, IOP and CCT alterations, the pairwise comparison before and after using the IOP-lowering medications was done by paired t-test. We performed Pearson or Spearman’s correlation analysis, as appropriate, to evaluate the relationship between the AST and ocular factors, including untreated IOP, axial length, VF parameters, and RNFL thickness. A *P* value < 0.05 was considered statistically significant, unless the Bonferroni correction method for multiple comparisons was applied, in which case a* P* < 0.025 was considered significant.

## Data Availability

The datasets generated during and/or analysed during the current study are available from the corresponding author on reasonable request.
